# The Negative Impact of COVID-19 in HCV, HIV, and HPV Surveillance Programs During the Different Pandemic Waves

**DOI:** 10.3389/fpubh.2022.880435

**Published:** 2022-07-22

**Authors:** Beatriz Romero-Hernández, Laura Martínez-García, Mario Rodríguez-Dominguez, Javier Martínez-Sanz, Manuel Vélez-Díaz-Pallarés, Belen Pérez Mies, A. Muriel, Francisco Gea, María Jesús Pérez-Elías, Juan Carlos Galán

**Affiliations:** ^1^Microbiology Department, Hospital Universitario Ramón y Cajal, Madrid, Spain; ^2^Instituto Ramón y Cajal for Health Research (IRYCIS), Madrid, Spain; ^3^CIBER of Epidemiology and Public Health (CIBERESP), Madrid, Spain; ^4^Department of Infectious Diseases, Hospital Universitario Ramón y Cajal, Madrid, Spain; ^5^CIBER of Infectious Diseases (CIBERINFEC), Madrid, Spain; ^6^Pharmacy Department, Hospital Universitario Ramon y Cajal, Madrid, Spain; ^7^Pathology Department, Hospital Universitario Ramón y Cajal, Madrid, Spain; ^8^Clinical Biostatistics Unit, Hospital Universitario Ramón y Cajal, Madrid, Spain; ^9^Universidad de Alcalá, Madrid, Spain; ^10^Liver Department, Hospital Universitario Ramón y Cajal, Madrid, Spain; ^11^CIBER of Liver and Digestive Diseases (CIBEREHD), Madrid, Spain

**Keywords:** impact of COVID-19, chronic viral infections, epidemic waves, decrease in HCV/HIV detection, lost infections

## Abstract

**Background:**

The coronavirus disease 2019 (COVID-19) pandemic has been a worldwide stress test for health systems. 2 years have elapsed since the description of the first cases of pneumonia of unknown origin. This study quantifies the impact of COVID-19 in the screening program of chronic viral infections such as human papillomavirus (HPV), human immunodeficiency virus (HIV), and hepatitis C virus (HCV) along the six different pandemic waves in our population. Each wave had particular epidemiological, biological, or clinical patterns.

**Methods:**

We analyzed the number of samples for screening of these viruses from March 2020 to February 2022, the new infections detected in the pandemic period compared to the previous year, the time elapsed between diagnosis and linking to treatment and follow-up of patients, and the percentage of late HIV diagnosis. Moreover, we used the origin of the samples as a marker for quantifying the restoration of activity in primary care.

**Results:**

During the first pandemic year, the number of samples received was reduced by 26.7, 22.6, and 22.5% for molecular detection of HPV or serological HCV and HIV status respectively. The highest decrease was observed during the first wave with 70, 40, and 26.7% for HPV, HCV, and HIV. As expected, new diagnoses also decreased by 35.4, 58.2, and 40.5% for HPV, HCV, and HIV respectively during the first year of the pandemic. In the second year of the pandemic, the number of samples remained below pre-pandemic period levels for HCV (−3.6%) and HIV (−9.3%) but was slightly higher for HPV (8.0%). The new diagnoses in the second year of the pandemic were −16.1, −46.8, and −18.6% for HPV, HCV, and HIV respectively.

**Conclusions:**

Undoubtedly, an important number of new HPV, HCV, and HIV infections were lost during the COVID-19 pandemic, and surveillance programs were disrupted as a consequence of collapse of the health system. It is a priority to reinforce these surveillance programs as soon as possible in order to detect undiagnosed cases before the associated morbidity-mortality increases. New pandemic waves could increase the risk of reversing the achievements made over the last few decades.

## Introduction

In March 2020, many countries implemented a strict lockdown to avoid the spread of severe acute respiratory syndrome coronavirus 2 (SARS-CoV-2) and the collapse of their public health systems (1). The lockdown and near collapse of hospitals affected the health of many citizens (2). For instance, 22.8% of Spanish patients refused to attend their health centers for fear of contagion (3), but also the non-COVID activity in Spanish hospitals drastically decreased. For example, 31.8% of surgeries related to any type of cancer were canceled in Spain, resulting in 45,449 fewer surgeries than had been arranged (4).

The direct impact of coronavirus disease 2019 (COVID-19) on infectious diseases has also been analyzed, especially as it relates to surveillance programs (5, 6). The number of new diagnoses has decreased, which will probably affect the quality of life of these undiagnosed individuals (7). An international estimation suggests that around 800,000 new hepatitis C viruses (HCV) were missed, and that as a result there will be 72,300 excess deaths in the coming years (8). Cervical cancer screening based on molecular detection of human papillomavirus (HPV) was suspended, in many countries, mainly between April and June 2020. This action could increase the incidence of cervical cancer and precancerous lesions in the future (9). These breaks in surveillance programs will lead to a dramatic delay in the World Health Organization (WHO) objectives for 2030 (10). All available studies, taken together, report the impact of COVID-19 over the first pandemic wave, limiting our understanding of the full scope of damage caused by the pandemic. In fact, access to primary care is not yet back to normal, and successive pandemic waves have altered many activities in the hospital setting. This work aims to analyze the impact of COVID-19, during 2 years, on surveillance programs for the early detection of new human immunodeficiency virus (HIV) diagnosis, viremic HCV status, and high-risk HPV (hrHPV) patients in a tertiary hospital and its corresponding health area.

## Methods

We carried out a 24 month observational retrospective study to determine the number of HIV, HCV, and HPV-positive cases recorded during the COVID-19 pandemic (from March 2020 to February 2022). The results were compared to cases observed during the pre-pandemic period (from March 2020 to February 2022) in Ramon y Cajal University Hospital, Madrid, which has a reference laboratory for 20 primary care centers and sees to more than 650,000 individuals. Since March 2020, six waves of the COVID-19 pandemic were observed in Spain: the first (March-June 2020), second (August-November 2020), third (December 2020-February 2021), fourth (April-June 2021), fifth (July-September 2021), and sixth (December-2021- February 2022) (11). During the period between March 2020 and February 2022, the Spanish government established several different social and health measures ranging from lockdown and a national state of alarm to a relaxation of such measures, which could have affected the diagnosis of non-COVID infectious diseases and surveillance programs differently. In accordance with the changing social and clinical measures, we have divided the pandemic into three phases. The first phase corresponds to the strict lockdown (corresponding with the highest mortality rate) and includes the first wave. The second phase includes the pre-vaccine period and the national state of alarm period until May 2021. The second and third waves occurred in this phase. The third phase is the period of vaccination of the population (including booster vaccinations prior to the sixth pandemic wave), the end of the national state of alarm and the progressive relaxation of social restrictions until their elimination (with the exception of masks in closed spaces). Unfortunately, three pandemic waves took place in this phase. These reasons are sufficient to assess the impact of pandemic waves in the surveillance programs for testing hrHPV, HCV, and HIV independently. We also analyzed the impact according to the origin of the samples in primary care or hospital settings. Blood and urine cultures were used as surrogate markers for relative quantifying of the negative impact of COVID-19 in the laboratory daily routine and to evaluate the re-establishment of regular activity at Ramón y Cajal University Hospital of in-hospital and out-of-hospital activities respectively.

The screening of cervical cancer based on the hrHPV detection is undertaken in sexually active women ranging between 25 and 65 years old, according to national guidelines. The detection of hrHPV is made in cervical liquid-based cytology. HIV and HCV tests were requested in serum samples from people with high-risk behaviors of expo-sure to these viruses in agreement with national screening programs^1^^,^^2^. These analyses require serum samples. As quality markers, we measured the time elapsed between diagnosis and the linking of treatment and follow-up with a specialized physician in pre-pandemic and pandemic periods. Moreover, we identified the percentage of late HIV diagnosis (<350 CD4/μL) in both periods.

For statistical analysis, the comparison between the number of tests requested in the different phases was made through a one-way ANOVA taking the year 2019 as a reference and adding Sidak correction for multiple comparisons. Non-parametric tests, Kruskal-Wallis test and Mann-Whitney tests were used for sensitivity analysis (12).

## Results

**Figure 1:**
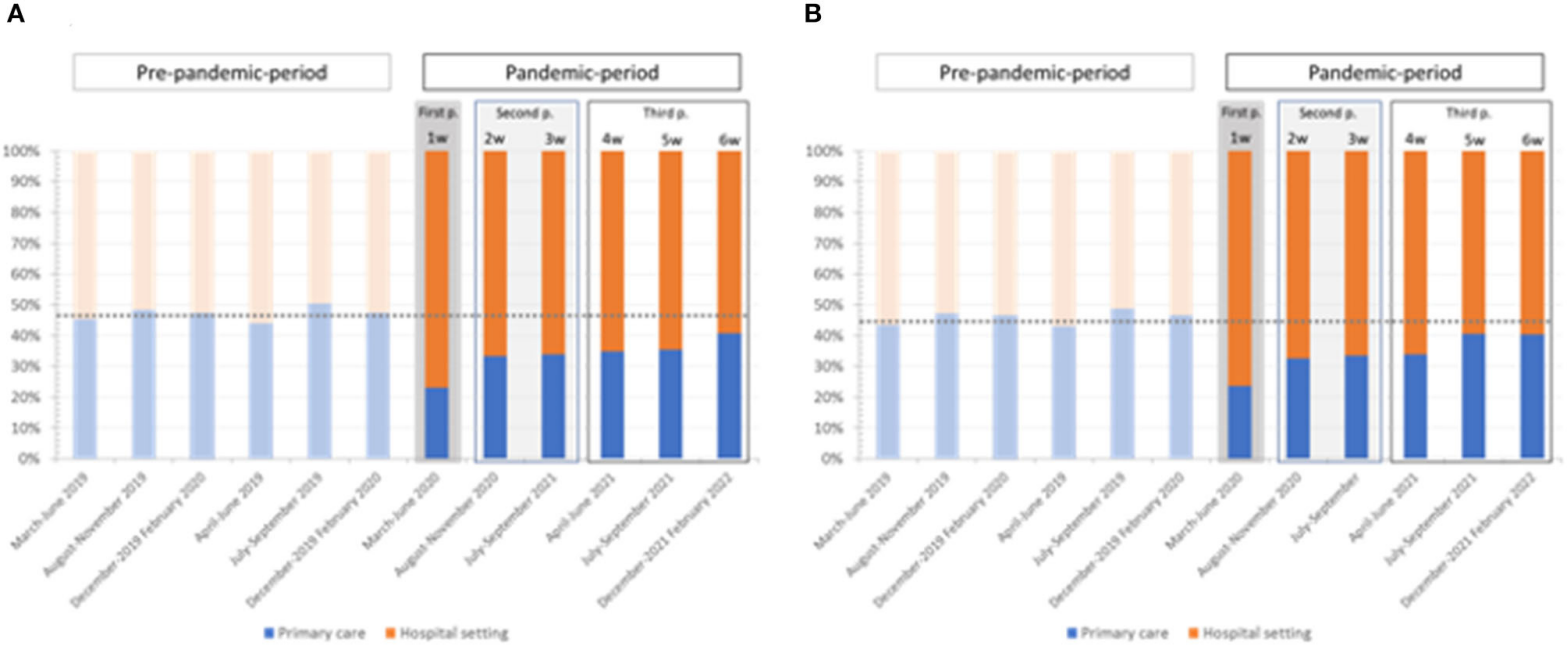
Percentage of samples received for HCV **(A)** and HIV **(B)** screening according to primary care or hospital setting origin. Dashed gray lines represent the mean percentage in pre-pandemic for samples from primary care (47% for HCV and 45% for HIV).

Overall, throughout the pandemic period studied (March 2020 to February 2022), the number of samples received by the Microbiology Department as the laboratory of reference for molecular detection of hrHPV or serological HCV and HIV status were 733 (−26.7%), 6,600 (−22.6%) and 6,919 (−22.5%) samples lower than 2019 respectively, during the 12 first months. These reductions were higher in phase I, 774 (−70.5%, fewer samples received), 4,814 (−40%) and 2,930 (−26.7%) for hrHPV, HCV and HIV respectively (Table 1). In phase II (corresponding to the second and third pandemic waves), the number of samples increased, although they were still lower than the pre-pandemic period for HCV and HIV (1,786 and 3,988, corresponding to −8.9 and −20.1% respectively). In the next 12 months (March 2021 to February 2022), the number of hrHPV samples was slightly higher (106 samples) corresponding to 3.9% more than in the pre-pandemic period. In HCV and HIV, the number of samples received remained lower than in the pre-pandemic period in 1,902 (−5.9%) and 3,429 (−11.0%) respectively. This second pandemic year corresponds to phase III (and three pandemic waves). For HCV, only in the sixth and last pandemic wave, was the number of samples slightly higher than in the pre-pandemic period (305 more samples received representing a 4% increase) whereas for HIV the “normal activity” was not recovered (Table 1). The surrogate markers used in this study showed that in-hospital activity (blood cultures) was recovered from the second phase, whereas the out-of-hospital activity in primary care (urine culture) did not recover until the third phase (Supplementary Figure 1). Although, the surrogate marker of primary care activity suggests a normal activity, when we compared the samples received according to the origin (Figure 1), we cannot conclude the restoration of “normal activity” for screening programs, because the number of samples from primary care is lower than the pre-pandemic period. The statistical analysis (Sidak correction for multiple comparison) indicates significant differences between phase I and the pre-pandemic period for all infections analyzed (*p* < 0.0001) for hrHPV and HCV and *p* < 0.006 for HIV. In the successive phases, we only found significant differences in phase II for HIV (*p* < 0.03).

**Table 1:** HPV (a), HCV (b) and HIV (c) determinations in pre-pandemic and pandemic period during the different pandemic waves.

	**First pandemic year**		**Second pandemic year**									
	**First phase**		**Second phase**						**Third phase**			
	**First wave**		**Second wave**		**Third wave**		**Fourth wave**		**Fifth wave**		**Sixth wave**	
	**Pre-P**	**Pand**	**Pre-P**	**Pand**	**Pre-P**	**Pand**	**Pre-P**	**Pand**	**Pre-P**	**Pand**	**Pre-P**	**Pand**
**(a) HPV**												
Number of analyzed samples	1097	323	1000	885	650	806	840	982	660	632	650	708
Difference of analyzed samples (%) in pandemic waves	−70.5%		−115 (−11.5%)		156 (24.0%)		142 (16.9%)		−28 (−4.2%)		58 (8.9%)	
Difference of analyzed samples (%) in pandemic phases	–**774 (**–**70.5%)**		**41 (2.5%)**				**172 (8.0%)**					
Infected patients	544	165	489	388	101	179	165	195	198	130	351	274
Difference of new patients (%) in pandemic waves	−70%		−20%		70%		20%		−35%		−22%	
Difference of new patients (%) in pandemic phases	–**379 (**–**69.6%)**		–**23 (**–**3.9%)**				–**115 (**–**16.1%)**					
**(b) HCV**												
Number of analyzed samples	12047	7233	12378	9415	7650	8827	9074	8836	8722	7733	7650	7955
Difference of analyzed samples (%) in pandemic waves	−40%		−2963 (−23.9%)		1177 (15.3%)		−238 (−2.6%)		−989 (−11.3%)		305 (4.0%)	
Difference of analyzed samples (%) in pandemic phases	–**4814 (**–**40.0%)**		–**1786 (**–**8.9%)**				–**922 (**–**3.6%)**							
Viremic patients	57	17	64	28	13	9	40	12	13	14	26	16
Difference of viremic patients (%) in pandemic waves	−70%		−56%		−30%		−70%		7%		−38%	
Difference of viremic patients (%) in pandemic phases	–**40 (**–**70%)**		–**38 (**–**49.3%)**				–**37 (**–**46.8%)**							
**(c) HIV**												
Number of analyzed samples	10986	8056	11460	8830	8339	6981	8288	7877	8214	7001	8339	7652
Difference of analyzed samples (%) in pandemic waves	−26.7%		−2630 (−22.9%)		1358 (−16.3%)		−411 (−4.9%)		−1213 (−14.8%)		−687 (−8.2%)	
Difference of analyzed samples (%) in pandemic phases	–**2930 (**–**26.7%)**		–**3988 (**–**20.1%)**				–**2311 (**–**9.3)**					
New infected patients	19	11	31	20	19	9	16	9	16	14	11	12
Difference of new patients (%) in pandemic waves	−42.1%		−35.5%		−52.6%		−43.7%		−12.5%		9.1%	
Difference of new patients (%) in pandemic phases	–**8 (**–**42.1%)**		–**21 (**–**42.0%)**				–**8 (**–**18.6%)**					
CI^*^/CM^**^	**~** **20/** **~** **250**		**~** **40/** **~** **100**		**~** **60/** **~** **150**		**~** **20/** **~** **25**		**~** **50/** **~** **35**		**~** **240/** **~** **60**	

*Phases indicate the public health measures; whereas waves correspond to exponential increase of COVID-19 cases in our population (see main text). Pre-P and Pand, correspond to pre-pandemic and pandemic period*.

* *(CI) Cumulative incidence, per 100,000 inhabitants*.

** *(CM)cumulative mortality per 1,000,000 inhabitants in the different pandemic waves (see text footnote 2)*.

**Figure 2:**
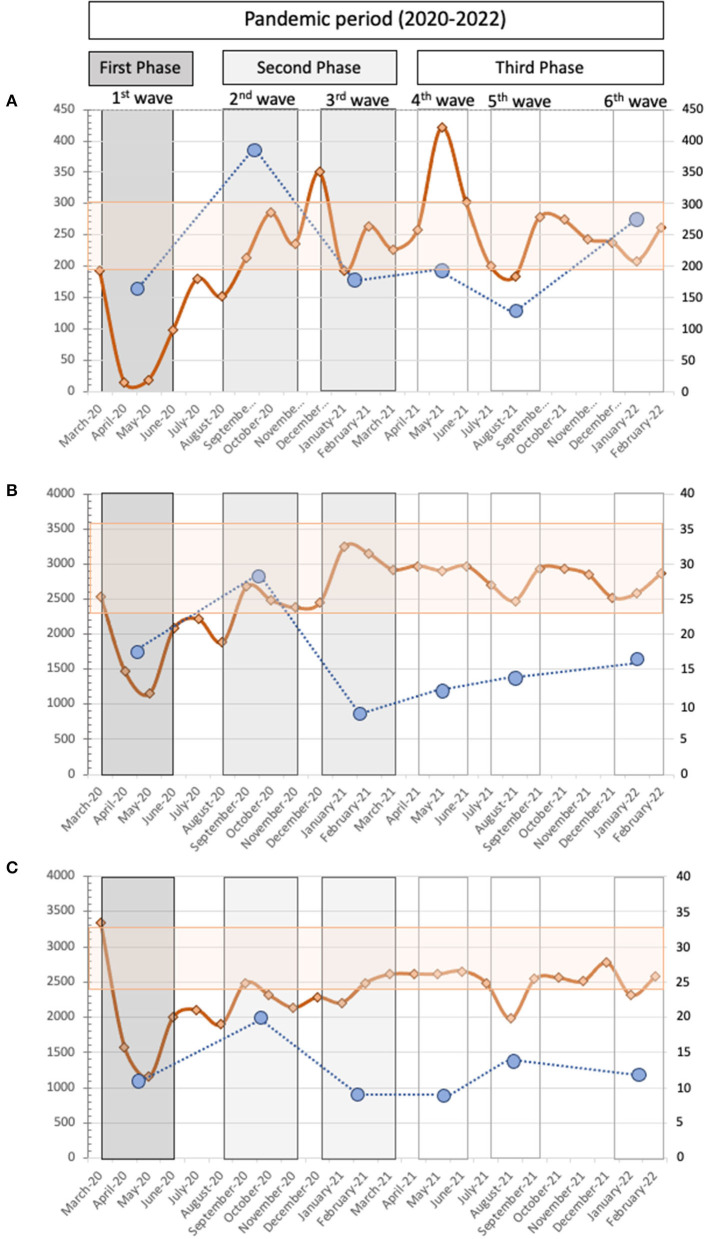
The monthly distributions of samples analyzed along the pandemic period for **(A)** HPV, **(B)** HCV, and **(C)** HIV are represented by orange solid lines. The upper/lower limit of samples/months obtained in pre-pandemic period are represented as light orange horizontal stick. The right y-axis corresponds to number of samples analyzed. The left y-axis corresponds to new diagnoses. Blue circles correspond to hrHPV infected patients **(A)**, viremic HCV patients **(B)**, or positive serological status against HIV **(C)**, in no previously known patients, in each pandemic wave (I–VI).

As to the new diagnoses detected during the first year of the pandemic, we also observed an important reduction of 402 (−35.4%), 78 (−58.2%), and 29 (−42.0%) patients for hrHPV, HCV, and HIV respectively. These reductions were higher in phase I, reaching −70% for hrHPV and HCV and −42.1% for HIV. In the second year of the pandemic (corresponding to phase III) 115 (−16.1%), 37 (−46.8%), and 8 (−18.6%) new diagnoses were lost for hrHPV, HCV, and HIV respectively (Figure 2). The statistical analysis (Sidak correction for multiple comparison) indicates significant differences between phase I and phase II compared to the pre-pandemic period for hrHPV (*p* < 0.0001) and HCV (*p* < 0.03), whereas no statistically significant differences were found in newly diagnosed cases of HIV among the different phases and the pre-pandemic period.

The most worrying findings were the delay in HIV diagnosis and the time elapsed between diagnosis and the linking to treatment. In 2019, the percentage of patients with <350 CD4/μL in the first analytical study post-HIV diagnosis was 36%. However, in 2021 this data reached 40%, and in the first months of 2022, around 66% cases were late HIV diagnoses (10/15 patients). As to the time elapsed between diagnosis and linking to treatment, this doubled from 24 to 48 days.

## Discussion

We have analyzed the impact of the COVID-19 pandemic on the diagnosis of chronic viral infectious diseases during a period of 24 months in a tertiary hospital in Spain, which was among the most seriously affected countries during the first pandemic wave between March-June 2020. Spain also had the highest percentage of vaccinated population from mid-June 2021 (13) and the booster dose was higher than the European average in February-22 ^3^. Between both periods (March 2020 to February 2022), up to six epidemic waves have been described, but obviously, the impact of the successive waves on health has been different. During the first three pandemic waves, corresponding to the first and second phases, (lockdown and pre-vaccination period) in the first year of the pandemic, the cumulative mortality at 14-days (CM) was always >100 cases/1,000,000 inhabitants. In the last three pandemic waves, corresponding to the third phase (vaccination including booster dose and progressive elimination of social measures) in the second year of the pandemic, CM at 14-days was <60 cases/1,000,000 inhabitants, even though the cumulative incidence (CI) was around 240/100,000 inhabitants in the sixth (and last) pandemic wave (11).

Our results show that the most drastic impact was during the first wave and corresponded to a strict lockdown period in which access to primary care centers was minimal, and the population's fear of COVID infection was widespread. This data suggests that the strict lockdown was a desperate solution with profound consequences both in the active screening programs for the viruses included in this study, and in the time elapsed between diagnosis and the linking of treatment and follow-up. In the first phase, the samples tested, and new diagnoses were reduced by around 70% for hrHPV. In the surveillances for HCV and HIV, the reduction of samples tested was lower than new infections detected (40% and 70% for HCV; 26.7% and 42.1% for HIV respectively). The observed decrease in HCV and HIV testing was lower than the number of requested HPV tests, because all patients who attended the Emergency Department (ED) for SARS-CoV-2 infection were also tested for HIV and HCV, which was not directly related to screening programs but rather to clinical decisions (14). This national recommendation did not increase the new number of diagnoses which was probably because the target population was not the most suitable for detecting new HIV and HCV, as the profile was older patients, the most susceptible group to hospital complications caused by COVID-19.

When the social restrictions and fear of contagion in the population decreased, the activity progressively recovered, and the number of samples analyzed increased during the second phase (second and third waves). It was not the same for the viruses analyzed in the successive waves. These differences could be attributed to the organization of our public health system. Gynecological activity among specialty centers began at the end of the first wave with pregnant women and those patients with altered cytology. In contrast, the infectious disease specialists were the last to return to their “normal activities” as they were responsible for managing COVID-19 patients. The “normal activity” for screening HPV, HCV and HIV was almost recovered by the third phase (fourth-sixth waves). These findings are related to the surrogate markers used in this study to quantify the primary care and hospital activities, but the number of samples for HCV and HIV screening from primary care was not completely recovered (the majority of new diagnoses are detected in primary care).

In our study, the number of infected patients was significantly lower than in the pre-pandemic year. There is an unknown number of infected people who could be lost for many years. In the following years, we should be able to measure the real impact of the COVID-19 pandemic. An estimation could be inferred based on our data in a single center. In 2 years (March 2020 to February 2022), we could have lost around 517, 115 and 37 new diagnoses of hrHPV, HCV and HIV respectively. Several studies suggest that a decrease in the hrHPV, HCV, HIV detection after the pandemic could lead to higher cancer mortality (15, 16) and a lower life expectancy for AIDS-defining conditions (17) in the following years. The delay in the HIV diagnosis is associated with non-infectious comorbidities and multimorbidity and contributes to higher total care costs (7). The percentage of late HIV diagnoses did not change drastically between 2019 and 2021 (36% and 40%). These values are consistent with national data (18); however, in the first months of 2022 (data not shown), the percentage of late diagnoses reached a worrying 66% (10/15 new diagnoses). This finding confirms our worst predictions: the loss of the efforts of health professionals' campaigns to reduce late HIV diagnoses over the last few years (19). A multidisciplinary group of American researchers suggested a rapid return to the pre-pandemic level, and that there would be minimal pandemic consequences related to HCV outcomes (20).

However, the simulations studies could be highly alarming and overrated. In many developed countries the new HIV diagnosis is declining due to pre-exposure prophylaxis (PreP) strategy (21), the vaccine against HPV infection or the micro-elimination programs, reducing the pool of viremic HCV patients drastically. In fact, a reduction in new HIV and HCV diagnoses was described in Spain during the years prior to COVID-19. In Madrid, around 38 and 44% fewer new HIV and HCV diagnoses were detected in the previous 2 years (22)^4^. These results suggest that those simulations must be interpreted with caution.

Nevertheless, positive consequences have been reached during the COVID-19 pandemic. Among them, digital technologies offer new opportunities to improve people's health with telemedicine (including treatment or telepharmacy) (24, 25). In our hospital, we have seen that our Pharmacy Department successfully applied telepharmacy to outpatient care during the COVID-19 pandemic and has distributed 13,084 shipments of treatment to these chronic patients to avoid them having to go to the hospital. This has contributed to minimizing the impact of COVID-19 as has been described (23).

We also understand that as the decentralizing in SARS-CoV-2 detection helped to control the pandemic, the decentralization in HIV/HCV diagnoses and care and the incorporation of telemedicine in high-resource settings will probably be critical in healthcare systems in the future. Our health system will need time to identify these lost infections, and the active surveillance programs should be reinforced as soon as possible. The implementation of these measures could accelerate the identification of cases and facilitate the linking to our public health system. These new measures will be necessary for a long period and the COVID-19 pandemic may well drive these changes.

## Data Availability Statement

The original contributions presented in the study are included in the article/Supplementary Material, further inquiries can be directed to the corresponding author.

## Author Contributions

JCG designed and supervised the study. BR-H collected data and wrote the manuscript with support from JCG, LM-G, and MR-D. MA performed the statistical analysis. JM-S, MV-D-P, BP, FG, and MP-E discussed the results and provided critical feedback to contribute to the final manuscript.

## Funding

This study was supported by the Instituto de Salud Carlos III (Spain) co-financed by the European Development Regional Fund (A Way to Achieve Europe program (PI20/01397 and CB06/02/0053) and the Ramon y Cajal Institute for Health Research (IRYCIS) (COVID-19 Grant 2020/0154).

## Conflict of Interest

BR-H has received funding to attend conferences from Gilead and obtained financed project from Roche. LM-G has obtained financed project from Roche. JM-S reports personal fees from ViiV Healthcare, Janssen Cilag, Gilead Sciences, and MSD and non-financial support from ViiV Healthcare, Jannsen Cilag, and Gilead Sciences. MP-E has received funding to attend conferences, educational activities or advisory, as well as scholarships from the pharmaceutical companies Gilead, Janssen, Abbvie, MSD, and ViiV. JCG received personal fees as advisor from Gilead, Abbvie, and Roche, he obtained financed projects from MSD, Roche and Sistemas Genómicos. The remaining authors declare that the research was conducted in the absence of any commercial or financial relationships that could be construed as a potential conflict of interest.

## Publisher's Note

All claims expressed in this article are solely those of the authors and do not necessarily represent those of their affiliated organizations, or those of the publisher, the editors and the reviewers. Any product that may be evaluated in this article, or claim that may be made by its manufacturer, is not guaranteed or endorsed by the publisher.

## Footnotes

^1^ https://www.mscbs.gob.es/ciudadanos/enfLesiones/enfTransmisibles/sida/docs/GuiaRecomendacionesDiagnosticoPrecozVIH.pdf

^2^https://www.sanidad.gob.es/ciudadanos/enfLesiones/enfTransmisibles/sida/docs/GUIA_DE_CRIBADO_DE_LA_INFECCION_POR_EL_VHC_2020.pdf (accessed July 2020).

^3^ https://ourworldindata.org/grapher/covid-vaccine-booster-doses-per-capita?time=2021-08-01.0.2022-02-21

^4^ https://www.comunidad.madrid/servicios/salud/datos-medicamentos-hepatitis-c
