# Supplementary figures and images for: The Negative Impact of COVID-19 in HCV, HIV, and HPV Surveillance Programs During the Different Pandemic Waves
Source: Front Public Health. 2022 Jul 22;10:880435. doi: 10.3389/fpubh.2022.880435 (PMC9353175; doi:10.3389/fpubh.2022.880435)

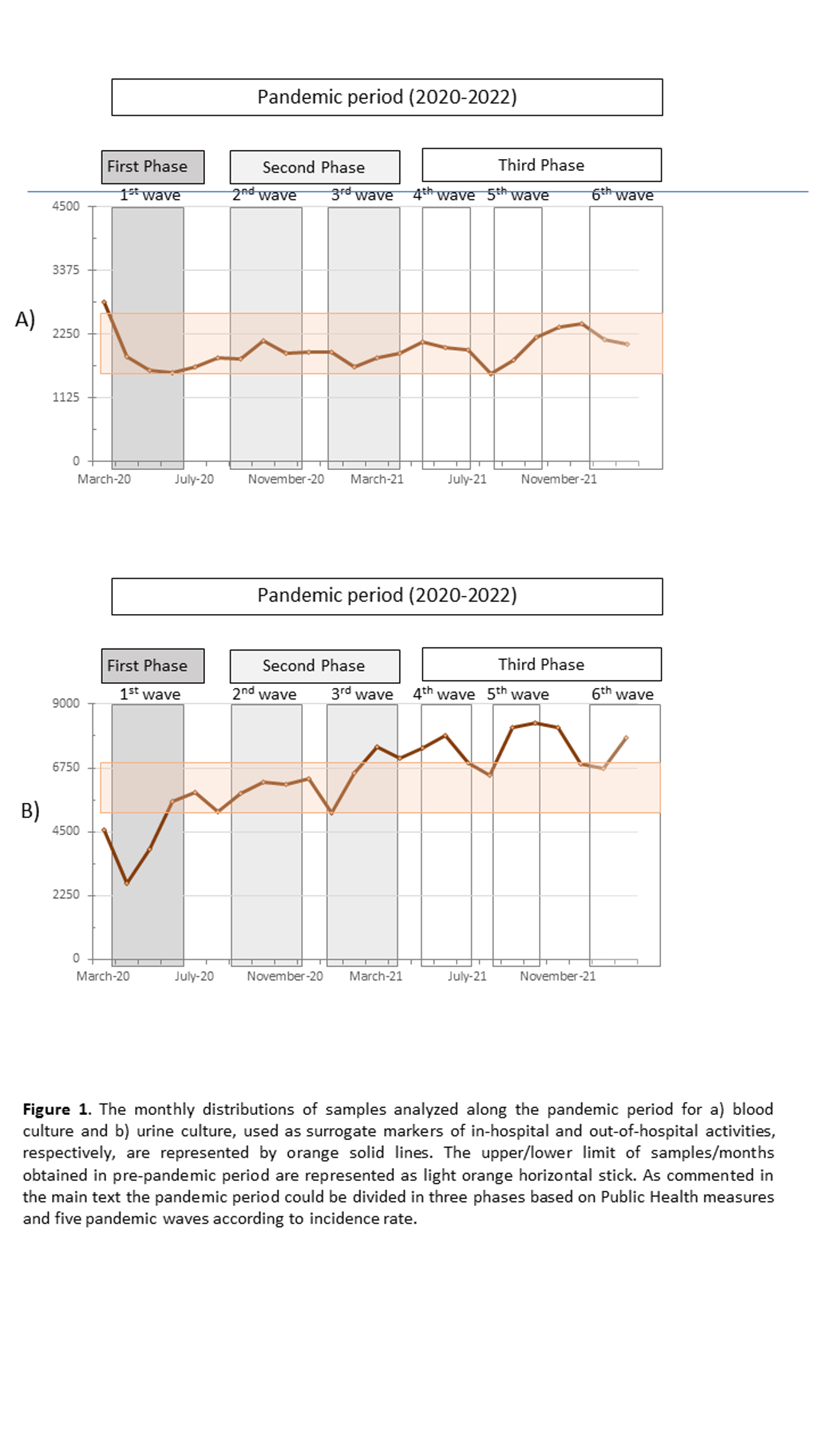

Supplement: Supplementary file 1 [file Image_1.tif]
